# Radiomics-based optimization of target selection in CT-guided percutaneous lung cancer biopsy: a retrospective study

**DOI:** 10.3389/fonc.2025.1701146

**Published:** 2026-01-15

**Authors:** JiWu Wang, ZeMing Zhang, XiaoDong Liu, XinYu Wang, YaoKang Chen, Jin Liu

**Affiliations:** 1 Radiological Imaging Department, Panzhihua Central Hospital, Panzhihua, Sichuan, China; 2 Radiological Imaging Department, Jianyang Chinese Medicine Hospital, Chengdu, Sichuan, China

**Keywords:** CT-guided biopsy, diagnostic yield, lung cancer, radiomics, target selection

## Abstract

**Background:**

CT-guided percutaneous transthoracic needle biopsy (PTNB) is a cornerstone diagnostic procedure for lung cancer. However, its diagnostic accuracy is frequently compromised by sampling errors arising from tumor heterogeneity and operator-dependent target selection, leading to false-negative outcomes. This study aimed to develop and validate a clinical–radiomics model based on biopsy-slot regions of interest (ROIs) to preoperatively predict tumor-rich targets and improve the diagnostic yield of CT-guided PTNB.

**Methods:**

In this retrospective study, a cohort of 350 patients with surgically confirmed lung cancer who underwent CT-guided PTNB was analyzed. Patients were classified into true-positive group (TPG) and false-negative group (FNG) based on pathological results and randomly allocated into training and validation sets. Radiomic features were extracted from standardized biopsy-slot ROIs, and feature selection was performed using least absolute shrinkage and selection operator (LASSO) regression. Independent clinical predictors were identified from a comprehensive set of candidate variables, including patient demographics, lesion characteristics, procedural factors, and classical lung cancer risk factors, using multivariate logistic regression and integrated with radiomic features to develop a combined prediction model. Model performance and clinical utility were assessed using receiver operating characteristic (ROC) and decision curve analysis (DCA).

**Results:**

Multivariate analysis identified age and vascular proximity (<0.5 cm) as the only independent clinical predictors of diagnostic success from among the candidate factors evaluated. The radiomics signature comprised 10 robust features derived from first-order statistics, neighboring gray tone difference matrix (NGTDM), gray level run length matrix (GLRLM), gray level size zone matrix (GLSZM), and wavelet transforms. The combined clinical–radiomics model demonstrated superior discriminative performance, achieving AUCs of 0.942 and 0.926 in the training and validation cohorts, respectively, significantly outperforming both the clinical model (AUCs: 0.703 and 0.696) and the radiomics model alone (AUCs: 0.883 and 0.867). ROC analysis established an optimal radiomics score (Rad score) cutoff of 0.42 (corresponding to a nomogram score of ≈165), yielding sensitivities of 89.6%–88.9% and specificities of 86.3%–84.7%, providing a clinically applicable threshold for biopsy target prioritization. The ROC curves visually confirm the performance of all three models.

**Conclusions:**

The proposed biopsy-slot ROI-based clinical–radiomics model accurately predicts tumor-rich targets in CT-guided PTNB for lung cancer. By synergistically integrating quantitative imaging biomarkers with key clinical variables, this model facilitates personalized biopsy planning and promotes precision-guided sampling strategies, potentially reducing nondiagnostic procedures. However, because this retrospective single-center study only included patients who subsequently underwent surgical resection, the findings may not be directly generalizable to inoperable patients or the broader population undergoing CT-guided PTNB.

## 1 Introduction

Lung cancer remains a major global health challenge, with recent data from China revealing it accounts for 22.0% of all cancer cases and 28.5% of cancer-related mortality (1, 2). This malignancy typically progresses asymptomatically in its early stages, resulting in over 70% of patients being diagnosed at advanced phases when curative interventions are no longer viable (3). Consequently, establishing accurate early diagnosis and initiating timely treatment are crucial for improving patient outcomes.

The implementation of low-dose computed tomography (LDCT) screening in high-risk populations has substantially improved the detection of small pulmonary nodules (4). However, radiological features alone frequently lack sufficient specificity to reliably distinguish benign from malignant lesions, making histopathological confirmation essential for definitive diagnosis (5). CT-guided percutaneous transthoracic needle biopsy (PTNB) has emerged as the preferred diagnostic modality in clinical practice, valued for its minimally invasive nature, targeting precision, and cost-effectiveness (6, 7). Although PTNB demonstrates a reported sensitivity exceeding 90% for lung cancer detection, real-world diagnostic accuracy ranges from 60% to 85%, primarily due to false-negative results caused by insufficient tumor cellularity in biopsy specimens (8–10). For optimal molecular profiling and personalized therapy planning, biopsy samples should ideally provide a tissue area exceeding 60 mm² with at least 20% tumor cell content (11). Current PTNB target selection relies heavily on the operator’s subjective interpretation of CT images and is further complicated by tumor heterogeneity, lesion characteristics, and anatomical considerations, all of which may compromise specimen quality and increase the risk of nondiagnostic outcomes (12).

Radiomics has emerged as a promising solution to these limitations by providing a quantitative, high-throughput framework for extracting multidimensional features from medical images. By analyzing texture, shape, and gray-level matrix patterns, radiomics captures intratumoral heterogeneity that reflects underlying biological behavior (13–15). In lung cancer, this approach has demonstrated considerable potential for genomic characterization, treatment response prediction, and outcome prognosis, indicating strong clinical translatability.

In this retrospective study, we analyzed 350 patients who underwent CT-guided PTNB with subsequent surgical confirmation of lung malignancy. Departing from conventional radiomics approaches that analyze entire tumor volumes, we introduced a novel, procedure-specific paradigm focusing on the biopsy cutting-slot area—the precise region of tissue acquisition. This targeted region of interest (ROI) strategy enables more accurate correlation between imaging features and pathological yield, effectively bridging quantitative radiomic analysis with clinical biopsy outcomes. By integrating radiomic features from this defined ROI with key clinical predictors, we developed and validated three predictive models—a radiomics model, a clinical model, and a combined model—to quantitatively identify tumor-rich regions, optimize target selection, and ultimately improve the diagnostic yield of CT-guided lung biopsies.

## 2 Materials and methods

### 2.1 Study population and data collection

This single-center, retrospective study was approved by the Institutional Review Board. We reviewed the records of 350 consecutive patients who underwent CT-guided PTNB for lung lesions at Panzhihua Central Hospital between October 2020 and April 2023. The final diagnosis of lung cancer for all included patients was pathologically confirmed by subsequent surgical resection. Because subsequent surgical resection was required for inclusion, the study cohort predominantly comprised patients with resectable disease and adequate performance status. Consequently, our sample does not fully capture the entire spectrum of patients undergoing CT-guided PTNB, particularly those with advanced or medically inoperable lung cancer in whom biopsy may be the sole source of tissue for diagnosis and molecular testing.

The inclusion criteria were (1) histopathologically confirmed lung cancer via surgical specimen; (2) preoperative CT-guided PTNB performed at our institution; (3) target lesion diameter greater than 1.5 cm; (4) biopsy core specimen length exceeding 1.0 cm; and (5) availability of complete clinical and imaging data.

The exclusion criteria included (1) significant deviation (> 0.5 cm) of the actual biopsy needle trajectory from the pre-planned target region; (2) pure ground-glass opacity nodules; and (3) incomplete or technically inadequate imaging datasets for radiomic analysis.

Based on the concordance between PTNB and surgical pathology results, patients were classified into two groups: the true positive group (TPG, n = 300), where PTNB correctly identified malignancy, and the false negative group (FNG, n = 50), where PTNB failed to diagnose the malignancy later confirmed by surgery. The entire cohort was then randomly split into a training set (70%, n = 245; 210 TPG, and 35 FNG) and an internal validation set (30%, n = 105; 90 TPG, and 15 FNG) using stratified sampling to preserve the ratio of TPG to FNG cases in both subsets.

We systematically collected the following clinical variables, identified from literature and clinical experience as potentially influencing biopsy yield: patient demographics (sex and age), lesion characteristics [maximal diameter on axial CT and composition (solid *vs*. mixed-density)], procedural anatomical factors (presence of high-risk puncture factors defined as vascular structures within 0.5 cm of the planned needle tract and intrapulmonary needle distance), and post-procedural complications (pneumothorax and bleeding).

In addition to age and vascular proximity, several classical lung cancer risk factors were also extracted from the electronic medical records, including smoking status (never, former, and current), cumulative smoking exposure (pack-years), history of chronic obstructive pulmonary disease (COPD), family history of lung cancer in first-degree relatives, and documented long-term occupational exposure to dust or chemical agents.

Complications were graded as follows:

- Pneumothorax: None, mild (<10% lung collapse), moderate (10%–30%), or severe (>30%).
- Bleeding: Graded by the maximum diameter of perilesional exudation: none, minimal (<2 cm), clinically significant (2–4 cm), or life-threatening (>4 cm) (16).

### 2.2 CT image acquisition protocol

All CT-guided localization and biopsy procedures were performed using a Philips Brilliance large-aperture CT scanner. A standardized protocol was used for all patients: tube voltage 120 kV, automated tube current modulation (200–300 mA), and acquisition of 1-mm isotropic slices with a table feed of 2 mm per rotation. Pre-procedural planning non-contrast CT images, which served as the source for all subsequent radiomic feature extraction, were obtained under consistent parameters during quiet respiration to minimize respiratory motion artifacts.

### 2.3 Biopsy procedure and specimen handling

All PTNB procedures were performed by one of three attending radiologists, each possessing over 5 years of specialized experience in thoracic intervention. The biopsy trajectory was meticulously planned on contrast-enhanced CT scans to target the largest solid component of the lesion while deliberately avoiding areas of visible necrosis, ribs, intercostal vessels, bullae, emphysematous regions, and major vascular structures or cavities within the projected cutting slot.

With the patient in an appropriate comfortable position, intravenous access was established, and vital signs were continuously monitored. After local infiltration anesthesia was administered down to the pleural surface, an 18-gauge automated or semi-automated biopsy needle was advanced incrementally under CT guidance along the pre-planned path to the target. A minimum of two core tissue samples were obtained from each lesion.

Immediately after the procedure, the obtained specimens were visually inspected independently by two experienced radiologists for macroscopic adequacy. Assessment criteria included specimen integrity (absence of excessive fragmentation), and the estimated proportion of anthracotic (carbon-pigmented) lung tissue, with samples containing less than 50% considered preferable. Specimens measuring longer than 1 cm with preserved gross morphological features were deemed adequate for routine pathological processing and subsequent analysis (17, 18).

### 2.4 Image registration and ROI segmentation

**Figure 1:**
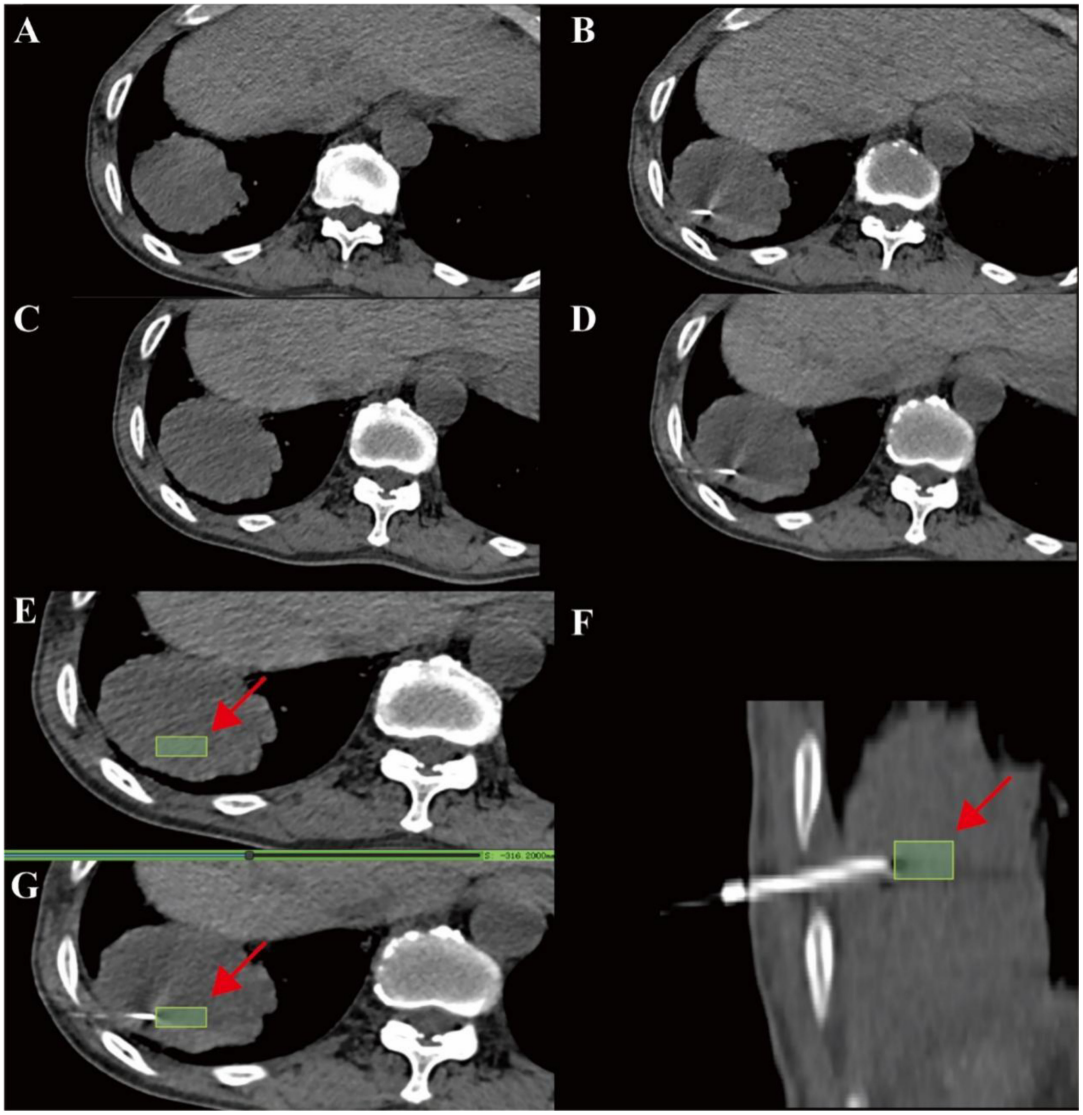
Registration of the pre-biopsy localization and intra-procedural cutting CT images. **(A)** Pre-biopsy planning CT scan. **(B)** Intra-procedural CT illustrating respiratory-related positional differences. **(C)** Fused image after registering **(A, B)**. **(D)** Reference image for analysis. **(E)** Axial CT image with manually delineated regions of interest (ROI) around the biopsy cutting slot (green box and red arrow). **(F)** Sagittal view of the biopsy needle path indicating the ROI location. **(G)** CT image during biopsy, showing the puncture needle track overlapping with the delineated ROI at the sampling site.

Image registration and segmentation were performed using the open-source software 3D Slicer (https://www.slicer.org/). To correct spatial misalignment caused by respiratory motion during biopsy, rigid registration was applied between the biopsy acquisition images (fixed images) and the preprocedural planning images (moving images) using the general registration [ Advanced Normalization Tools (ANTS)] module in 3D Slicer, following the standard principles of medical image registration (19). The registration and fusion of the pre-biopsy planning CT and intra-procedural cutting CT images show the pre-biopsy scan (**Figure 1A**), the respiratory-related positional differences during the procedure (**Figure 1B**), the fused image obtained after the rigid registration (**Figure 1C**), and the reference image used for analysis (**Figure 1D**).

Before segmentation, all Digital Imaging and Communications in Medicine (DICOM) images were anonymized and intensity-normalized using the Medical Image Preprocessing Toolkit (v0.1.4) to reduce scanner-related heterogeneity (20). ROIs were manually delineated on the registered planning CT images by Physician A (7 years of experience in PTNB and thoracic image interpretation), referencing the confirmed biopsy needle tract. Each ROI was defined as a three-dimensional cuboid volume centered on the biopsy cutting slot, with fixed dimensions of 1.5 cm (length along the needle tract) × 0.5 cm (width) × 1.0 cm (depth, covering two 0.5-cm slices above and below the target point), and aligned with the biopsy trajectory. To further ensure that the cuboid approximated the actual tissue sampled by the needle, the location and orientation of each ROI were reviewed on axial, sagittal, and coronal planes and, when necessary, manually adjusted so that the long axis of the cuboid followed the biopsy track and the volume encompassed the expected cutting window of the needle. For smaller lesions or lesions adjacent to critical structures, particular care was taken to minimize obvious extension of the cuboid into large vessels, pleura, or aerated normal lung parenchyma as far as reasonably achievable. By design, however, the fixed-size cuboid ROI may still contain a thin rim of perilesional tissue, which reflects the clinical reality that core needle specimens often include a mixture of lesional and adjacent non-lesion tissue. To assess reproducibility, 50 randomly selected cases underwent repeat segmentation by Physician A after a 1-month interval and independent segmentation by Physician B (4 years of experience), and segmentation stability and interobserver agreement were quantified using the Dice similarity coefficient (scale 0–1, with scores near 1 representing better overlap) (**Figures 1E–G**).

### 2.5 Radiomic feature extraction and preprocessing

Feature extraction was performed using the PyRadiomics package (v3.0.1) integrated within the 3D Slicer, strictly adhering to the Image Biomarker Standardization Initiative (IBSI) guidelines to ensure reproducibility (21). A critical preprocessing step was applied to all ROIs before feature calculation: isotropic voxel resampling (to 1 × 1 × 1 mm³) and intensity discretization using a fixed bin width of 25 Hounsfield Units (HU). This step is essential for mitigating the influence of varying scan parameters and enhancing the biological interpretability of texture features by reducing noise and standardizing the intensity scale across the cohort (21, 22).

A total of 1,023 radiomic features were extracted from each standardized ROI, which were categorized as follows:

- First-order statistics (18 features): describing the distribution of voxel intensities within the ROI (e.g., mean, variance, skewness, and kurtosis).
- Second-order and higher-order texture features (75 features): quantifying intra-regional heterogeneity patterns, including features from:
  ○ Gray level co-occurrence matrix (GLCM, 24 features)
  ○ Gray level run length matrix (GLRLM, 16 features)
  ○ Gray level size zone matrix (GLSZM, 16 features)
  ○ Gray level dependence matrix (GLDM, 14 features)
  ○ Neighboring gray tone difference matrix (NGTDM, 5 features)
- Wavelet-filtered features (930 features): Obtained by applying eight different wavelet decompositions (LLL, LLH, LHL, LHH, HLL, HLH, HHL, and HHH) to the original image and extracting the first-order and texture features from each filtered output, thereby capturing multi-scale texture information.

Notably, all shape-based features (e.g., volume, sphericity, and compactness) were explicitly excluded from the analysis. Since the ROIs were predefined as uniform cuboids aligned with the needle tract, any shape descriptors would solely reflect this artificial geometry rather than the intrinsic morphological properties of the underlying tissue, thus preventing the introduction of non-biological bias.

### 2.6 Feature selection and radiomics signature construction

To develop a robust and generalizable radiomics signature, we designed a multi-step feature selection and dimensionality-reduction pipeline explicitly guided by the principle of maximum relevance and minimum redundancy (mRMR). First, all 1,023 extracted features were standardized using Z-score normalization. The reproducibility of each feature was then assessed in 50 randomly selected cases that were segmented twice by Physician A and once by Physician B. Features with an intraclass correlation coefficient (ICC) < 0.75 for either intra- or inter-observer agreement were discarded, ensuring that the subsequent signature was built upon stable and reproducible measurements (23).

Second, to minimize redundancy among the remaining features, a Spearman correlation matrix was computed. For any pair of features with an absolute Spearman correlation coefficient (|ρ| > 0.85), the feature with the higher mean absolute correlation with all other features was iteratively removed, thereby minimizing redundancy (23). This correlation-based filtering step effectively reduced multicollinearity and retained a more parsimonious set of nonredundant descriptors, consistent with the “minimum redundancy” component of the mRMR framework.

Third, to maximize relevance to the clinical endpoint, the reduced feature set was entered into a least absolute shrinkage and selection operator (LASSO) logistic regression model with a 10-fold cross-validation. The optimal penalty parameter (λ) was selected by minimizing the mean cross-validated deviance, and only features with nonzero coefficients at this λ were retained. By shrinking the coefficients of weakly informative features toward zero and preserving those with the strongest association with true-positive biopsy outcomes, LASSO operationalized the “maximum relevance” component of the mRMR principle.

Taken together, this three-step strategy—reproducibility filtering, correlation-based redundancy reduction, and LASSO-based relevance selection—was designed to approximate an mRMR-guided radiomics signature that balances parsimony, robustness, and biological interpretability. The final radiomics signature consisted of 10 features, and an individual radiomics score (Rad-score) for each patient was calculated as a weighted linear combination of these features, with weights given by the corresponding LASSO coefficients.

The final set of 10 selected features comprised a combination of first-order statistics, wavelet-derived features, and texture features from NGTDM, GLRLM, and GLSZM. A radiomics score (Rad-score) was then calculated for each patient as a weighted linear combination of these selected features, with the weights being the coefficients derived from the LASSO regression model.

The formula for the Rad-score is:


$$Rad-score=\Sigma \;\left(Feature\_Value_{i}\;\times \;Coefficient_{i}\right)\;\text{for}\;\text{i}=1\;\text{to}\;10$$

This continuous Rad-score serves as an integrative index, where a higher value indicates a higher imaging-based probability that the sampled biopsy target contains sufficient tumor cells for a definitive diagnosis (i.e., a true-positive outcome).

### 2.7 Predictive model development and validation

Three distinct multivariate logistic regression models were constructed to predict the likelihood of obtaining a tumor-rich biopsy sample: (1) Clinical model: This model incorporated only the significant clinical predictors identified through our preliminary univariate and multivariate analyses. (2) Radiomics model: This model utilized the continuous Rad-score as the sole predictor variable. (3) Combined clinical–radiomics model: This integrated model included both the significant clinical predictors and the Rad-score to leverage their complementary predictive information.

To enhance clinical translatability and provide a practical tool for individualized risk estimation, a nomogram was constructed based on the final combined model. This graphical calculator allows clinicians to sum points assigned to each predictor (clinical factors and Rad-score) and project the total onto a probability scale indicating the chance of a successful, tumor-rich biopsy.

### 2.8 Model performance and clinical utility assessment

The discriminatory power of each model was rigorously evaluated in both the training and internal validation cohorts. Receiver operating characteristic (ROC) curves were generated, and the area under the curve (AUC) was calculated along with its 95% confidence interval (CI). Performance metrics, including sensitivity, specificity, and accuracy, were reported at the optimal probability cutoff determined by the Youden index.

Statistical comparison of the ROC curves between the different models was performed using DeLong’s test for correlated AUCs (24), to determine if improvements in performance were statistically significant.

Beyond traditional discrimination metrics, the clinical utility and potential impact of implementing these models were assessed using Decision curve analysis (DCA) (25). DCA estimates the standardized net benefit across a continuous range of threshold probabilities, thereby quantifying the clinical value of using a model to guide biopsy target selection (e.g., “biopsy this target” *vs*. “avoid this target”) compared to default strategies of conducting a biopsy of all lesions or not.

### 2.9 Statistical analysis

All statistical analyses were conducted using SPSS (version 26.0) and R (version 3.6.0, with packages including pROC for ROC analysis, Regression Modeling Decision Analysis (rmda) for DCA, and generalized linear models via penalized maximum likelihood (glmnet) for LASSO regression). A two-tailed P-value < 0.05 was considered statistically significant for all tests.

All candidate clinical variables were first evaluated in univariable logistic regression analyses with diagnostic biopsy outcome (diagnostic *vs*. non-diagnostic) as the dependent variable. Variables with P < 0.10 in univariable analysis and those considered clinically important *a priori* were subsequently entered into a multivariable logistic regression model. A backward stepwise selection procedure based on the Akaike information criterion was used to derive the final clinical model. Model performance was assessed using the area under the ROC curve (AUC), sensitivity, specificity, and calibration in both the training and validation cohorts.

The distribution of continuous variables was assessed for normality using the Kolmogorov–Smirnov test. Normally distributed data were summarized as mean ± standard deviation and compared between groups using independent-samples t-tests. Non-normally distributed data were presented as median with interquartile range (IQR) and compared using the Mann–Whitney U test. Categorical variables were expressed as frequencies and percentages, and group differences were evaluated using the Chi-square test or Fisher’s exact test, as appropriate.

For the primary model comparisons, the differences in AUCs between the clinical, radiomics, and combined models were formally tested for statistical significance using the DeLong’s test (24).

## 3 Results

### 3.1 Clinical data analysis

To establish a robust baseline for model development, we systematically evaluated a comprehensive set of clinical variables known or hypothesized to influence biopsy yield. These included patient demographics (sex and age), lesion characteristics (diameter and composition), procedural anatomy (high-risk puncture factors and intrapulmonary needle distance), and post-procedural outcomes (bleeding and pneumothorax). In addition, several classical epidemiologic risk factors for lung cancer were recorded, including smoking status (never, former, and current), cumulative smoking exposure (pack-years), history of COPD, family history of lung cancer, and documented occupational exposure to dust or chemical agents. The baseline characteristics of the training and internal validation cohorts are detailed in **Table 1**.

**Table 1:** Baseline characteristics and clinical features of the training and internal validation cohorts.

Characteristic	Training cohort (N = 245)	Validation cohort (N = 105)	P-value
Demographics			
Sex, male	144 (58.8%)	67 (63.8%)	0.371
Age, years	66.0 [54.0, 74.0]	64.0 [54.0, 73.0]	0.469
Lesion characteristics			
Diameter, cm	3.00 [2.30, 4.20]	3.30 [2.40, 4.80]	0.161
Composition			0.895
Solid	193 (78.8%)	82 (78.1%)	
Mixed density	52 (21.2%)	23 (21.9%)	
Procedural factors			
High-risk puncture factors*	146 (59.6%)	55 (52.4%)	0.2
Intrapulmonary needle distance, cm	1.30 [0.00, 2.70]	1.40 [0.40, 3.00]	0.562
Epidemiologic risk factors†			
Smoking status			0.95
Never	90 (36.7%)	40 (38.1%)	
Former	80 (32.7%)	32 (30.5%)	
Current	75 (30.6%)	33 (31.4%)	
Pack-years, among ever-smokers	30.0 [15.0, 45.0]	28.0 [15.0, 42.0]	0.48
COPD history, n (%)	52 (21.2%)	21 (20.0%)	0.8
Family history of lung cancer, n (%)	38 (15.5%)	15 (14.3%)	0.78
Occupational exposure, n (%)	41 (16.7%)	16 (15.2%)	0.72
Post-procedural complications			
Bleeding‡			0.607
None	184 (75.1%)	81 (77.1%)	
Mild	50 (20.4%)	23 (21.9%)	
Moderate	9 (3.7%)	1 (1.0%)	
Severe	2 (0.8%)	0 (0.0%)	
Pneumothorax§			0.662
None	189 (77.1%)	81 (77.1%)	
Mild	46 (18.8%)	20 (19.0%)	
Moderate	9 (3.7%)	3 (2.9%)	
Severe	1 (0.4%)	1 (1.0%)	

Data are presented as n (%) or median [interquartile range]. P-values were derived from the χ² test, Fisher’s exact test, or Mann–Whitney U test, as appropriate.

*High-risk puncture factors were defined as the presence of vascular structures within 0.5 cm of the planned biopsy trajectory.

†Epidemiologic risk factors included smoking status, pack-years among ever-smokers, COPD history, family history of lung cancer, and occupational exposure.

‡Bleeding was graded by the maximum diameter of perilesional exudation: none, mild (<2 cm), moderate (2–4 cm), and severe (>4 cm).

§Pneumothorax was graded as: none, mild (<10% lung collapse), moderate (10–30%), and severe (>30%).

**Figure 2:**
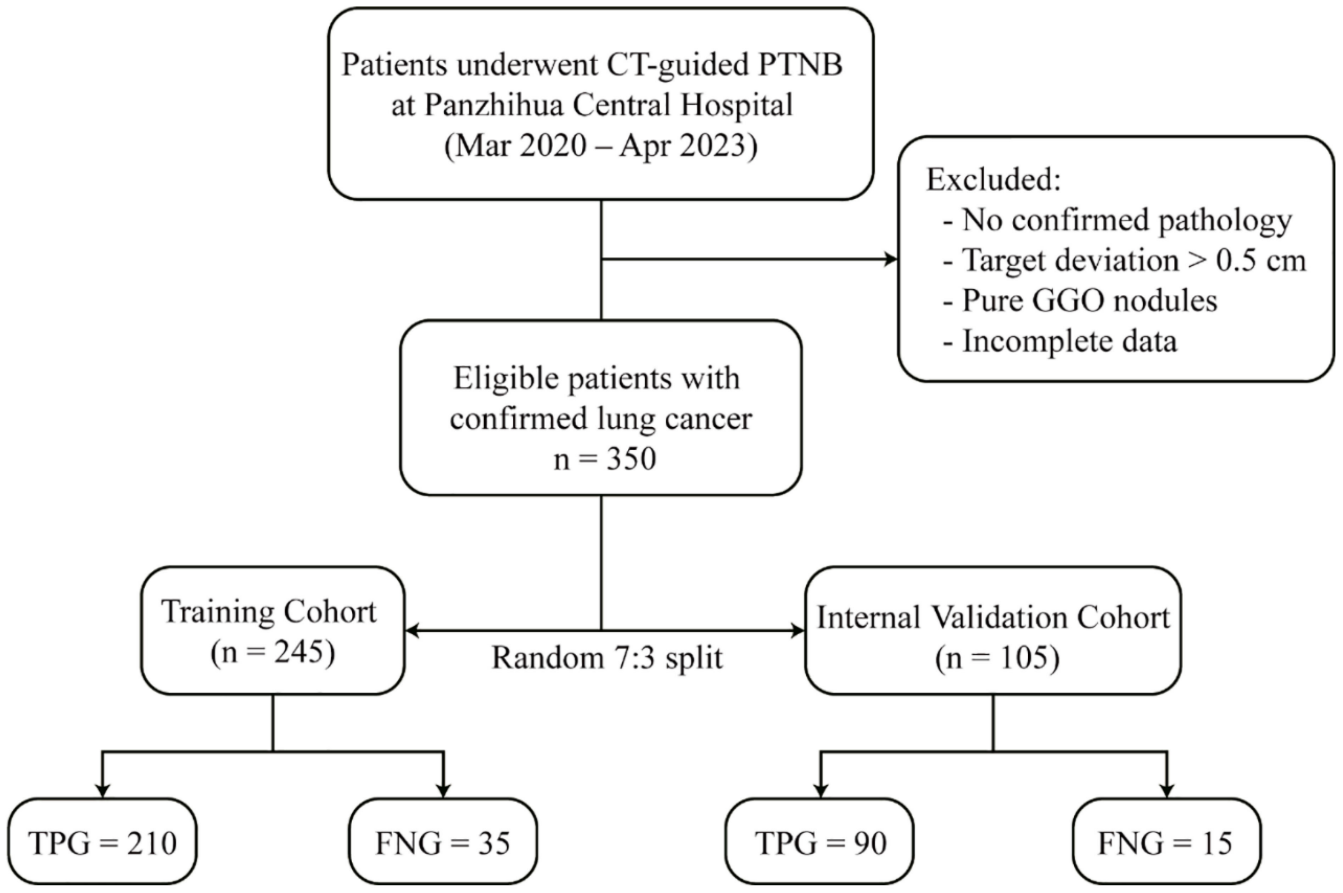
Patient selection and enrollment flowchart.

A total of 350 eligible patients who met all inclusion and exclusion criteria were ultimately enrolled for model development and validation. The cohort was randomly partitioned into a training set (n = 245, 70%) and an internal validation set (n = 105, 30%) using a stratified randomization approach to maintain a consistent ratio of positive to negative outcomes across both subsets. The training cohort comprised 210 TPG and 35 FNG cases, while the validation cohort contained 90 TPG and 15 FNG cases, accurately reflecting the overall disease prevalence in the study population. The complete patient selection pathway, from initial screening to final analysis, is delineated in **Figure 2**. Given the marked class imbalance in our dataset (300 TPG *vs*. 50 FNG cases), stratified sampling was used to preserve the original class distribution in both the training and validation cohorts. However, the relatively small number of FNG cases, particularly in the validation set (n = 15), inevitably limits the precision of performance estimates for the minority class. Consequently, the confidence intervals for the AUC and other metrics should be interpreted with caution, especially with regard to specificity.

### 3.2 Clinical model development

A comparative analysis of baseline characteristics revealed no statistically significant differences between the TPG and FNG in terms of sex, lesion diameter, intrapulmonary needle distance, lesion composition, or postoperative complications (all P > 0.05), as detailed in **Table 2**. However, two variables demonstrated significant associations with the biopsy outcome: patients in the TPG were significantly older than those in the FNG (median age: 67.0 years *vs*. 55.5 years, P = 0.005), and the TPG exhibited a significantly higher prevalence of high-risk puncture factors (60.0% *vs*. 42.0%, P = 0.026). These findings suggest that age and high-risk puncture factors might be important clinical determinants of biopsy yield. Accordingly, age, high-risk puncture factors, together with several classical epidemiologic risk factors (smoking status, cumulative smoking exposure, history of COPD, family history of lung cancer, and occupational exposure), were subsequently entered as candidate predictors in the univariable and multivariable logistic regression analyses used to construct the clinical model (**Table 3**).

**Table 2:** Comparison of baseline characteristics between the true-positive group (TPG) and false-negative group (FNG).

Variable	TPG (N = 300)	FNG (N = 50)	*P*-value
Sex, n (%)			0.295
Male	177 (59.0%)	34 (68.0%)	
Female	123 (41.0%)	16 (32.0%)	
Age (years)	67.0 [56.0, 74.0]	55.5 [45.5, 64.8]	0.00532**
Lesion diameter (cm)	3.04 [2.40, 4.43]	3.15 [2.10, 4.10]	0.528
High-risk puncture factors, n (%)			0.0261*
Yes	180 (60.0%)	21 (42.0%)	
No	120 (40.0%)	29 (58.0%)	
Intrapulmonary needle distance (cm)	1.30 [0.30, 2.70]	1.10 [0.00, 3.00]	0.523
Lesion composition, n (%)			0.506
Mixed density	62 (20.7%)	13 (26.0%)	
Solid density	238 (79.3%)	37 (74.0%)	
Postoperative bleeding, n (%)			0.651
None	225 (75.0%)	40 (80.0%)	
Mild	65 (21.7%)	8 (16.0%)	
Moderate	8 (2.7%)	2 (4.0%)	
Severe	2 (0.7%)	0 (0.0%)	
Postoperative pneumothorax, n (%)			0.296
None	226 (75.3%)	44 (88.0%)	
Mild	61 (20.3%)	5 (10.0%)	
Moderate	11 (3.7%)	1 (2.0%)	
Severe	2 (0.7%)	0 (0.0%)	

*P < 0.05, **P < 0.01 are considered statistically significant. Data are presented as median [IQR] or n (%).

**Table 3:** Univariate analysis of clinical factors associated with diagnostic biopsy.

Variable	OR	95% CI	P-value
Age (per 10 years)	1.58	1.20–2.10	0.001
Current smoker *vs*. never	1.95	1.10–3.46	0.022
Former smoker *vs*. never	1.32	0.76–2.28	0.320
Pack-years (per 10 pack-years)	1.21	1.07–1.37	0.003
History of COPD	2.34	1.15–4.76	0.019
Family history of lung cancer	1.45	0.80–2.64	0.220
Occupational exposure	1.62	0.87–3.02	0.130
Vascular proximity (yes *vs*. no)	2.80	1.60–4.90	<0.001

OR, odds ratio; CI, confidence interval. P < 0.05 is considered statistically significant.

In univariable analyses, older age (per 10-year increase: OR = 1.58, 95% CI: 1.20–2.10, P = 0.001), current smoking status (*vs*. never smoking: OR = 1.95, 95% CI: 1.10–3.46, P = 0.022), higher cumulative smoking exposure (per 10 pack-years: OR = 1.21, 95% CI: 1.07–1.37, P = 0.003), and history of COPD (OR = 2.34, 95% CI: 1.15–4.76, P = 0.019) were significantly associated with a higher likelihood of obtaining a diagnostic biopsy. Family history of lung cancer and occupational exposure showed a trend toward increased diagnostic yield but did not reach statistical significance (both P > 0.10). Detailed results of the univariable analyses are summarized in **Table 3**.

In the subsequent multivariable logistic regression that included the above candidate variables, only age and vascular proximity remained independent predictors of diagnostic biopsy outcomes (both P < 0.05), whereas smoking-related and other epidemiologic risk factors were not retained in the final clinical model.

### 3.3 Radiomic feature signature

From the initial set of 1,023 features extracted from the biopsy-slot ROIs, a final signature of 10 robust and nonredundant features was identified through the feature selection pipeline. The list of these features and their corresponding coefficients in the radiomics model are presented in **Table 4**. The radiomics score (Rad-score) derived from these features demonstrated a significant difference between the TPG and FNG in both the training and validation cohorts (P < 0.001).

**Table 4:** Radiomic features selected for puncture target characterization with corresponding coefficients.

Radiomic feature label	Coefficient	Feature type
original_firstorder_Kurtosis	-0.0257	First-order
original_firstorder_Skewness	0.1385	First-order
original_glrlm_ShortRunLowGrayLevelEmphasis	0.0835	GLRLM
original_ngtdm_Complexity	-0.0680	NGTDM
original_ngtdm_Strength	-0.1308	NGTDM
log.sigma.4.0.mm.3D_firstorder_Variance	-0.0368	First-order
log.sigma.4.0.mm.3D_glszm_SizeZoneNonUniformity	0.2157	GLSZM
wavelet. LLH_glrlm_LowGrayLevelRunEmphasis	0.0262	GLRLM
wavelet. HHL_glcm_ClusterShade	0.0168	GLCM
wavelet. LLL_glcm_InverseVariance	0.0275	GLCM

Coefficients indicate the weights derived from the least absolute shrinkage and selection operator (LASSO) logistic regression model.

**Figure 3:**
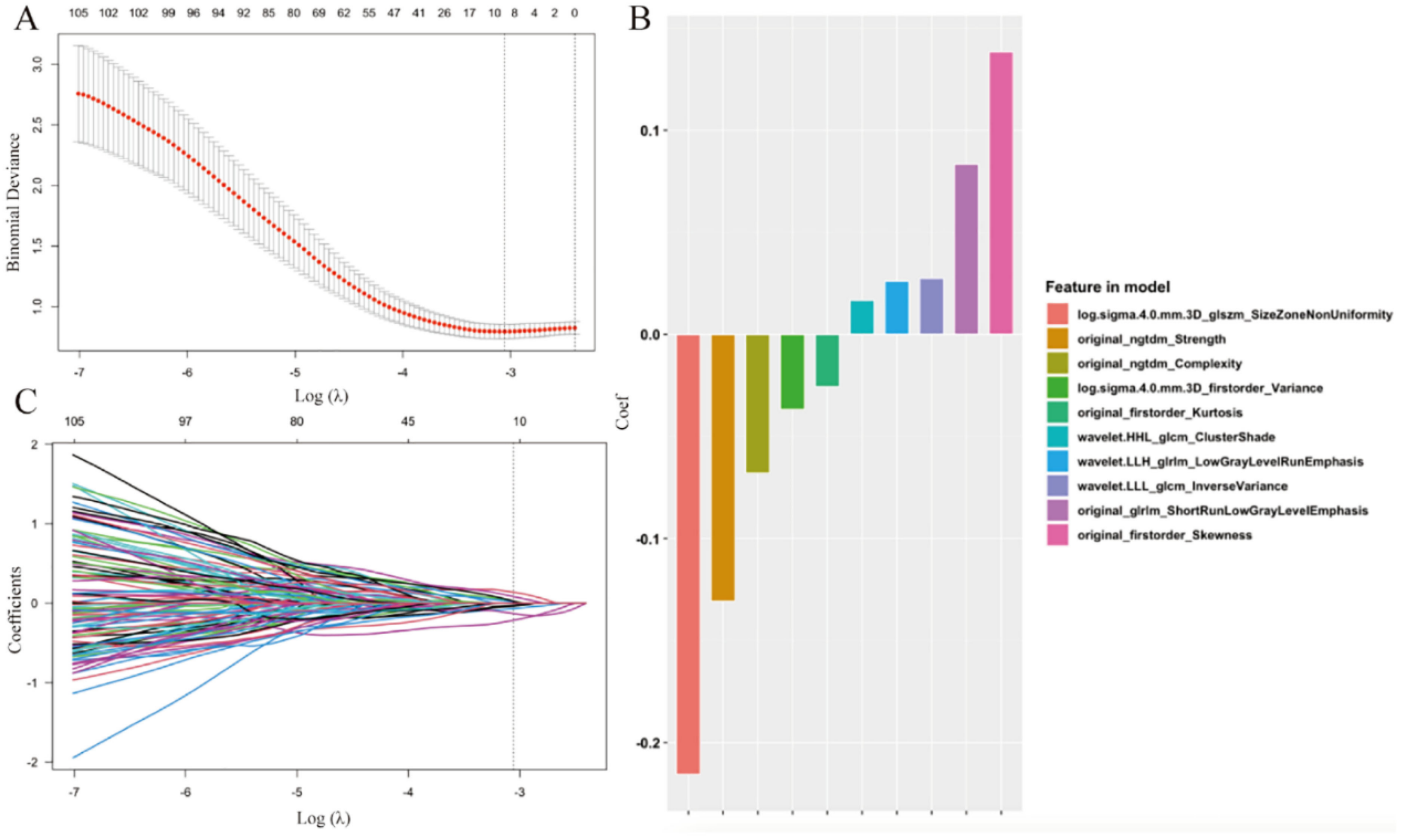
The least absolute shrinkage and selection operator (LASSO) feature selection and radiomics model coefficients. **(A)** The LASSO binomial deviance curve. Log(λ) is plotted along the x-axis, and mean square error (MSE) is shown on the y-axis. The upper x-axis represents the count of features preserved at each λ value. The vertical dashed line marks the optimal log(λ) that was chosen based on a five-fold cross-validation. **(B)** The bar plot of regression coefficient weights for the 10 radiomic features in the radiomics model. **(C)** The LASSO coefficient profile plot showing the shrinkage of feature coefficients as log(λ) increases. As regularization decreases, the top x-axis shows the count of features that remain.

As shown in **Figure 3**, the LASSO regression with a five-fold cross-validation identified the optimal penalty parameter at log(λ) = −3.13, corresponding to the minimum binomial deviance (**Figure 3A**). Ten features with nonzero coefficients were retained at this value, mainly derived from first-order statistics, gray-level size-zone and co-occurrence matrices, and multi-scale wavelet features. Their coefficients (**Figure 3B**) indicate each feature’s contribution to the radiomics model, while the coefficient path plot (**Figure 3C**) demonstrates progressive shrinkage as regularization increases. This process yielded a concise and robust model with reduced overfitting risk.

### 3.4 Diagnostic performance of the three models

**Figure 4:**
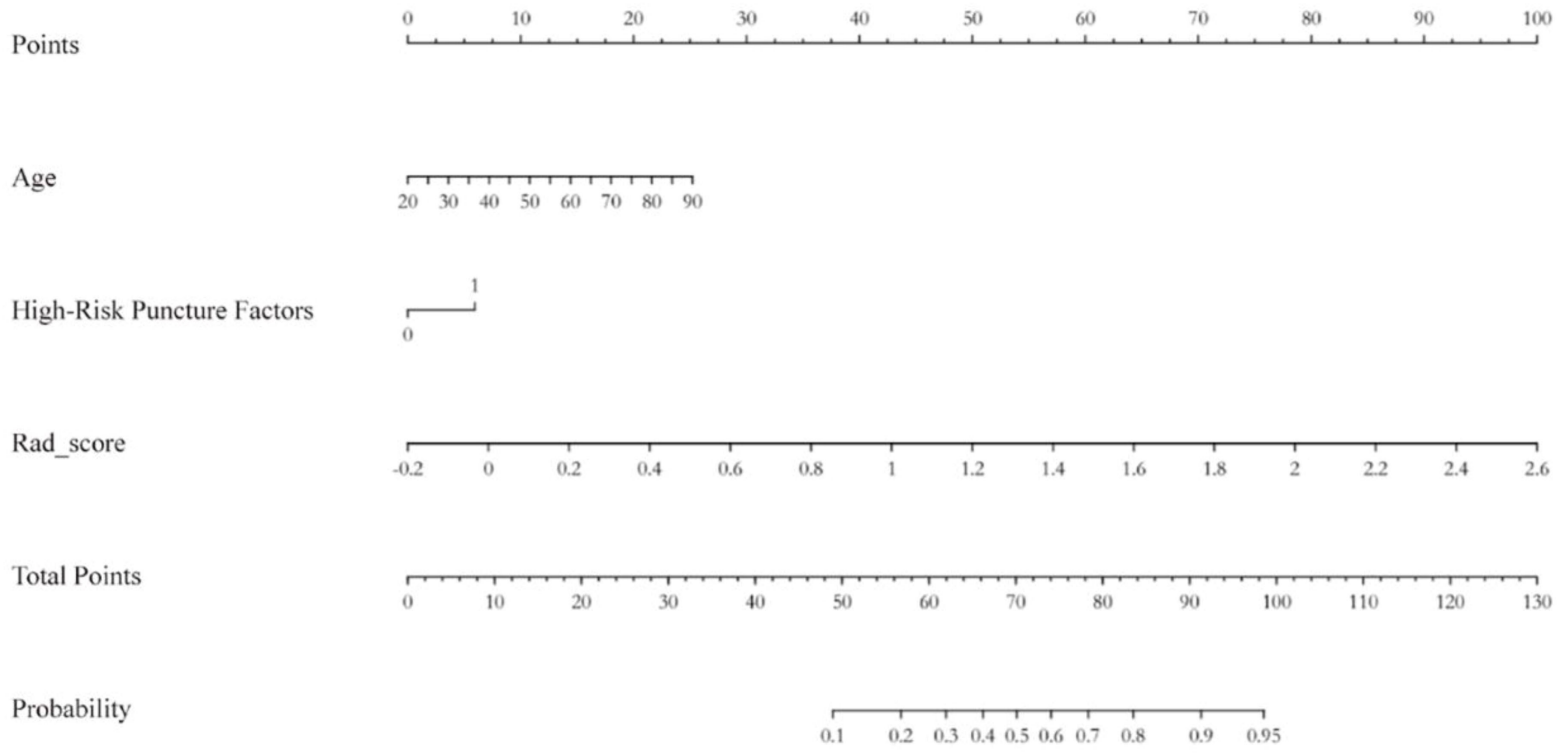
Predictive nomogram for the integrated clinical–radiomics model.

Based on the model construction framework, a nomogram was generated to visualize the combined clinical–radiomics model for individualized prediction of true positive biopsy targets (**Figure 4**).

The diagnostic performance of the clinical, radiomics, and combined models for identifying true positive biopsy targets is summarized in **Table 5**. As shown, the combined model achieved superior discriminative accuracy in both training and validation cohorts. For the training cohort, AUC values were 0.703 for the clinical model, 0.883 for the radiomics model, and 0.942 for the combined model. Consistent findings were observed in the validation cohort, with corresponding AUCs of 0.696, 0.867, and 0.926. Moreover, the combined model achieved higher sensitivity, specificity, and overall accuracy compared with the other two models in both cohorts.

**Table 5:** Predictive performance of the clinical, radiomics, and combined models for identifying true positive biopsy targets.

Model	Cohort	AUC (95% CI)	Sensitivity	Specificity	Accuracy
Clinical model	Training cohort	0.703 (0.615–0.791)	0.697	0.701	0.688
	Internal validation cohort	0.696 (0.527–0.866)	0.678	0.658	0.671
Radiomics model	Training cohort	0.883 (0.748–0.972)	0.856	0.843	0.823
	Internal validation cohort	0.867 (0.732–0.969)	0.844	0.844	0.865
Combined model	Training cohort	0.942 (0.865–0.999)	0.921	0.917	0.901
	Internal validation cohort	0.926 (0.830–0.999)	0.903	0.897	0.884

The 95%CI for the AUC in the validation cohort was relatively wide, reflecting the limited number of FNG cases (n = 15) and the resulting uncertainty in the estimation of model performance, particularly with respect to specificity.

**Figure 5:**
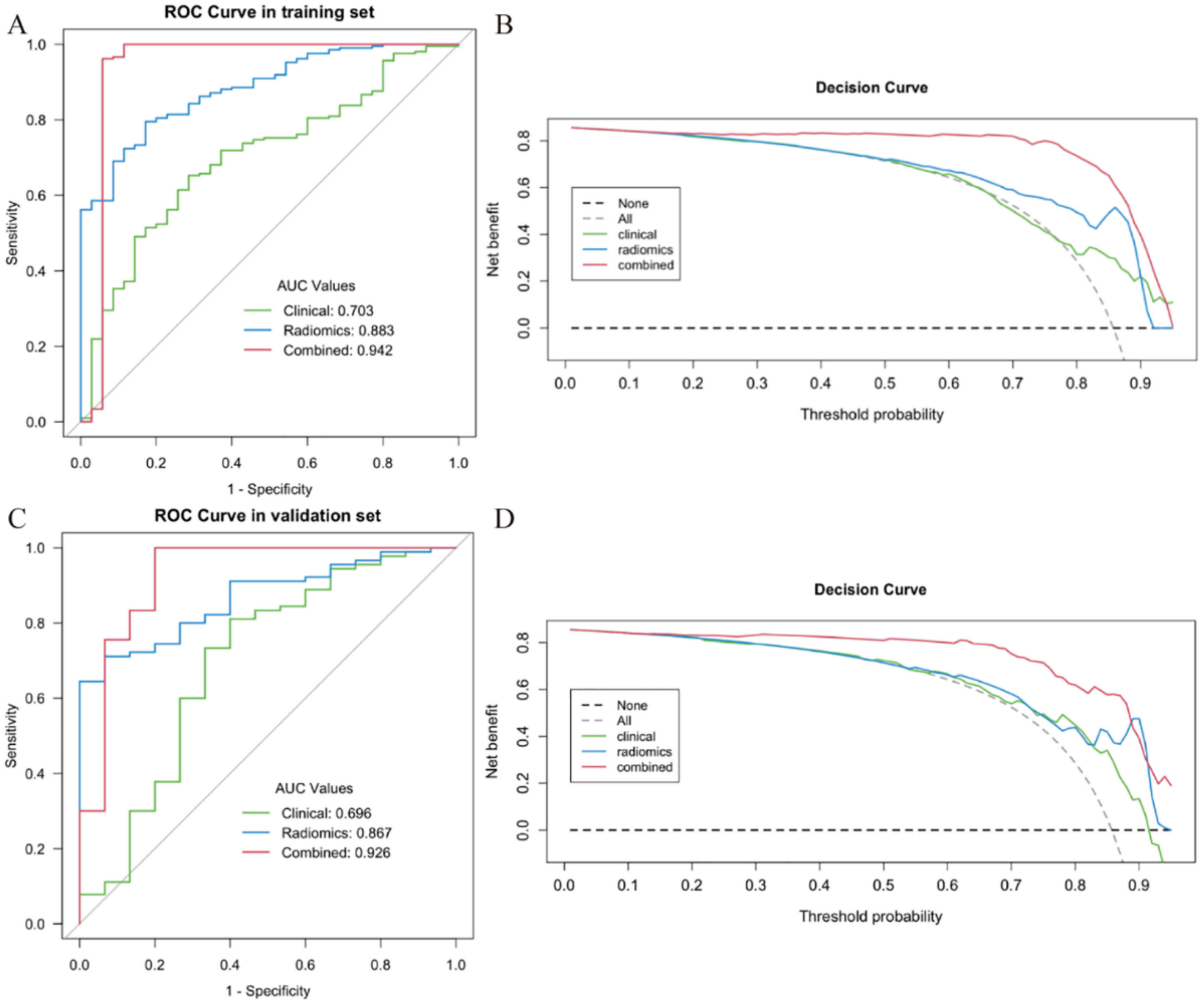
The performance of different models evaluated by the receiver operating characteristic (ROC) and decision curve analysis (DCA) in training and validation cohorts. **(A)** The ROC plot for the training cohort. **(B)** The DCA in the training cohort. The graph’s y-axis reflects net benefit, and its x-axis displays threshold probability. **(C)** The ROC plot for the internal validation cohort. **(D)** The DCA performed on the internal validation cohort.

The ROC curves (**Figures 5A, C**) visually confirmed that the combined model achieved the largest AUC in both cohorts, underscoring its superior predictive capability compared with either model alone. DeLong tests (**Table 6**) further indicated that both the radiomics model (P = 0.000451) and the combined model (P = 0.000024) significantly outperformed the clinical model. Furthermore, statistical analysis revealed that the combined model performed significantly better than the radiomics model alone (P = 0.018354), highlighting the incremental value of incorporating clinical parameters. In the decision curve analyses (**Figures 5B, D**), the combined model also demonstrated the highest net benefit across a broad range of threshold probabilities in both the training and validation cohorts, suggesting its superior clinical utility for guiding biopsy target selection.

**Table 6:** Statistical comparison of predictive performance among the different models for identifying true positive biopsy targets.

Model comparison	*P*-value
Clinical model *vs*. Radiomics model	0.000451***
Clinical model *vs*. Combined model	0.000024****
Radiomics model *vs*. Combined model	0.018354*

*P < 0.05, ***P < 0.001, and ****P < 0.0001 are considered statistically significant.

**Figure 6:**
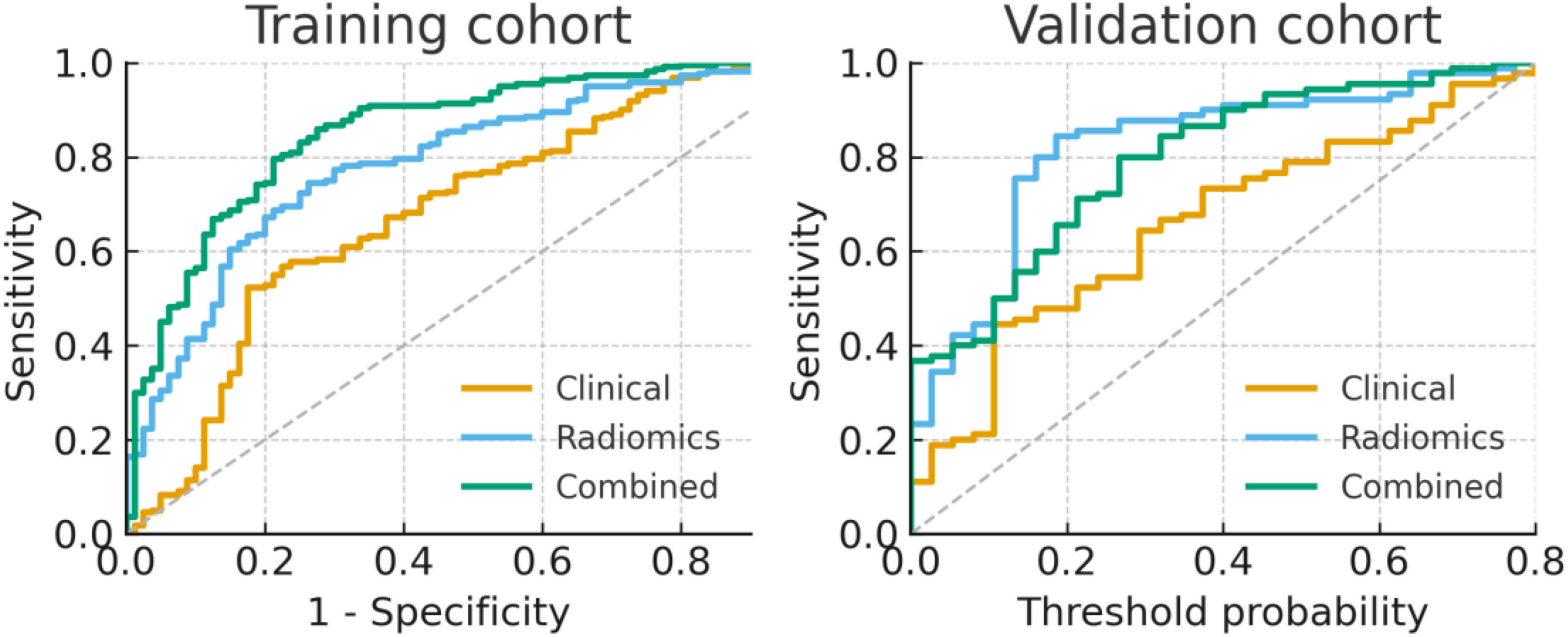
The receiver operating characteristic (ROC)-based determination of the optimal radiomics score (Rad-score) cutoff and comparison of the clinical, radiomics, and combined models in the training and validation cohorts. (Left) Overlaid ROC curves of the clinical, radiomics, and combined models in the training cohort, with the Youden index-derived optimal cutoff for the combined model (Rad-score = 0.42) indicated on the curve. (Right) Overlaid ROC curves of the three models in the internal validation cohort using the same Rad-score threshold, demonstrating a consistent classification performance.

To enhance clinical interpretability, ROC analysis was further used to determine an optimal Rad-score cutoff for classifying biopsy targets as likely “tumor-rich” versus “non-diagnostic.” As shown in **Figures 6A, B**, the ROC curves of the clinical, radiomics, and combined models are overlaid for the training and validation cohorts, respectively, with the Youden index-derived optimal cutoff for the combined model indicated on each plot. A Rad-score threshold of 0.42 achieved a sensitivity of 89.6% and a specificity of 86.3% in the training cohort. When the same cutoff was applied to the validation cohort, the corresponding sensitivity and specificity were 88.9% and 84.7%, respectively. In the nomogram derived from the combined model, this threshold corresponds to a total point score of approximately 165, which can serve as a practical reference for decision-making in clinical workflows.

## 4 Discussion

CT-guided PTNB maintains its pivotal role in the pathological diagnosis of pulmonary lesions, yet its diagnostic efficacy continues to be challenged by several fundamental limitations. As demonstrated in our cohort of 350 patients, where 50 cases (14.3%) yielded false-negative results, the procedure’s accuracy remains suboptimal despite technical advancements. This diagnostic shortfall primarily stems from tumor heterogeneity, operator-dependent target selection, and the inherent limitations of conventional CT interpretation in characterizing intralesional variability (26). Even with meticulous planning to avoid macroscopic necrosis on contrast-enhanced CT, subtle textural variations and the complex spatial architecture of viable tumor cells frequently lead to nonrepresentative sampling (27). While advanced imaging modalities, including PET/CT and MRI, offer superior metabolic and functional characterization, their implementation in routine biopsy planning remains constrained by cost considerations and limited availability in many clinical settings (28, 29).

In addressing these challenges, radiomics has emerged as a promising computational framework that enables comprehensive quantification of intratumoral heterogeneity through high-dimensional analysis of texture, intensity, and spatial relationships (30, 31). The methodological innovation of our study lies in the strategic focus on biopsy-slot specific ROIs, which precisely correspond to the actual tissue acquisition sites rather than encompassing the entire tumor volume. This approach represents a significant paradigm shift from conventional radiomics methodologies, as it establishes a direct correlation between imaging features and the specific tissue columns obtained during biopsy procedures (32). Our results substantiate the validity of this approach, with the radiomics model demonstrating substantially superior discriminatory capability (AUC: 0.883 in training, 0.867 in validation) compared to conventional clinical assessment alone.

Our multivariate analysis identified two independent clinical predictors of successful biopsy outcomes: patient age and vascular proximity. Although we systematically evaluated a set of classical epidemiologic risk factors for lung cancer—including smoking status, cumulative smoking exposure, history of COPD, family history of lung cancer, and occupational exposure—only age and vascular proximity remained independent predictors in the final multivariable clinical model. The significant age disparity between true-positive and false-negative groups (median: 67.0 *vs*. 55.5 years, P = 0.005) and the elevated prevalence of high-risk puncture factors in diagnostic samples (60.0% *vs*. 42.0%, P = 0.026) provide compelling clinical insights. These findings align with established oncological principles wherein perivascular regions typically exhibit enhanced tumor cellularity and viability, likely attributable to improved perfusion and nutrient delivery (33, 34). The moderate correlation observed between age and vascular proximity (r = 0.449) suggests potential age-related alterations in tumor microenvironment characteristics, though both variables maintained independent predictive significance. Notably, the absence of significant associations between biopsy yield and other procedural variables—including lesion diameter, density characteristics, and intrapulmonary needle distance—likely reflects the standardized technical approach employed, particularly the consistent use of 18-gauge needles and the exclusion of subcentimeter lesions (35, 36). Importantly, by combining reproducibility-based filtering, correlation-based redundancy reduction, and LASSO regression within an mRMR-guided framework, the resulting radiomics signature comprises a compact set of nonredundant, highly informative features that are more amenable to biological interpretation and external validation.

The radiomic signature derived through our rigorous feature selection process encompassed 10 stable features spanning multiple mathematical domains, including first-order statistics, gray-level matrices (NGTDM, GLRLM, and GLSZM), and wavelet-transformed parameters. This multi-scale feature ensemble captures complementary aspects of tissue heterogeneity, potentially reflecting underlying biological properties such as cellular density, necrotic fraction, and microvascular complexity. The integration of these quantitative imaging features with clinical predictors yielded a combined model achieving exceptional discriminative performance, with AUCs of 0.942 and 0.926 in the training and validation cohorts, respectively. Statistical comparisons confirmed the significant superiority of the combined model over both clinical-only and radiomics-only approaches (P < 0.05 for all comparisons), highlighting the synergistic value of integrating clinical and imaging data. It presents the ROC curves for all three models, and the combined model consistently achieved the largest AUC in both cohorts. While the clinical model alone demonstrated more modest discriminatory capability, it provides a valuable baseline, and its integration in the combined model contributed to a statistically significant performance improvement over the radiomics model alone.

For practical clinical implementation, we established an optimal Rad-score cutoff of 0.42, corresponding to a nomogram score of 165, which maintained balanced performance metrics across both cohorts (sensitivity >88% and specificity >84%). This threshold provides an objective, quantitative framework for interventional radiologists to prioritize biopsy targets based on their likelihood of yielding tumor-rich tissue. The clinical translation of this approach appears feasible through integration with existing workflow infrastructure. Utilizing open-source platforms such as the 3D Slicer, radiologists can efficiently extract standardized features from planned biopsy trajectories (21), while potential integration with picture archiving and communication systems (PACS) or CT guidance consoles could enable real-time assessment of target suitability prior to needle insertion. Such implementation holds promise for reducing non-diagnostic procedures, minimizing repeat biopsies, and optimizing tissue acquisition for molecular profiling—critical considerations in the era of precision oncology.

Several methodological limitations warrant careful consideration. First, the retrospective, single-center design inherently limits generalizability and may introduce both selection and spectrum bias, despite our rigorous inclusion criteria and standardized protocols. Because enrollment required histopathological confirmation of lung cancer on a subsequent surgical specimen, our cohort predominantly comprised patients with resectable disease and adequate performance status. This spectrum differs from the broader population undergoing CT-guided PTNB in routine practice, which includes a substantial proportion of inoperable or medically fragile patients in whom PTNB is the sole or primary source of tissue for diagnosis and molecular profiling. Therefore, the present model should be interpreted as being primarily applicable to patients with operable lung cancer or lesions judged to be amenable to surgery, and its performance in more advanced or nonsurgical cases remains uncertain. Prospective, multicenter studies that explicitly include nonsurgical and inoperable patients will be essential to validate the robustness and generalizability of our findings.

Second, the dataset was highly imbalanced, with substantially more TPG than FNG cases (300 *vs*. 50). Although we applied stratified splitting to preserve the original class distribution in the training and validation cohorts, the small number of FNG cases in the validation set (n = 15) reduces the statistical power to robustly estimate specificity and other performance metrics for the minority class. This is reflected by the relatively wide confidence intervals for the AUC (e.g., 0.830–0.999 for the combined model), indicating considerable uncertainty around the point estimates. As a result, the apparent discriminatory performance of the combined model in our validation cohort may overestimate its true performance in broader clinical populations, particularly for FNG cases. Future studies with larger and more balanced cohorts, or designs enriched for FNG lesions, are needed to more precisely characterize model performance for the minority class and to confirm the robustness of our findings.

Third, the biopsy-slot ROI was modeled as a fixed-size cuboid rather than an exact reconstruction of the true needle trajectory and sampled tissue volume. Although the cuboid was centered on the planned biopsy track and its position was visually verified by an experienced interventional radiologist, some degree of mismatch between the ROI and the actual tissue sampled is unavoidable, especially for smaller or irregularly shaped lesions. In such cases, the cuboid may partially extend beyond the lesion boundaries and encompass adjacent non-lesion tissue (e.g., atelectasis, vessels, or normal lung parenchyma). This partial-volume effect could dilute lesion-specific radiomic signals and introduce additional noise into the feature set, thereby contributing to variability in model performance. Future work incorporating more precise localization of the needle tip and track, adaptive ROI sizing, or deformable lesion segmentation may help better approximate the true biopsy volume and further refine the model.

Fourth, despite the overall high diagnostic yield of CT-guided PTNB in our cohort, the absolute number of false-negative cases was small, which further limited our ability to perform detailed subgroup analyses and more granular model refinement. Furthermore, while we incorporated several classical lung cancer risk factors into our clinical model analysis, only age and vascular proximity were retained as independent predictors. The role of other factors, such as genetic predisposition or more detailed environmental exposures, warrants investigation in larger, potentially prospective studies.

## 5 Conclusion

In conclusion, our study suggests that a clinically integrated radiomics approach, strategically focused on biopsy-slot specific ROIs, has the potential to enhance the prediction of tumor-rich targets in CT-guided lung biopsy. By simultaneously leveraging quantitative imaging heterogeneity and relevant clinical factors, this methodology provides a promising framework for refining biopsy planning and execution in patients with operable lung cancer. Although the internally validated performance of our model and its feasible implementation pathway support its potential utility for precision-guided sampling in thoracic oncology, prospective multicenter studies in broader PTNB populations, including inoperable patients and lesions with more diverse characteristics, are warranted before routine clinical adoption.

## Data Availability

The raw data supporting the conclusions of this article will be made available by the authors, without undue reservation.
